# Evaluation of peripartum supplementation of methionine hydroxy analogue on beef cow–calf performance

**DOI:** 10.1093/tas/txad046

**Published:** 2023-05-09

**Authors:** Colby A Redifer, Daniel D Loy, Curtis R Youngs, Chong Wang, Allison M Meyer, Heather A Tucker, Patrick J Gunn

**Affiliations:** Department of Animal Science, Iowa State University, Ames, IA 50011, USA; Department of Animal Science, Iowa State University, Ames, IA 50011, USA; Department of Animal Science, Iowa State University, Ames, IA 50011, USA; Department of Statistics, Iowa State University, Ames, IA 50011, USA; Department of Veterinary Diagnostic and Production Animal Medicine, Iowa State University, Ames, IA 50011, USA; Division of Animal Sciences, University of Missouri, Columbia, MO 65211, USA; Novus International Inc., St. Charles, MO 63304, USA; Department of Animal Science, Iowa State University, Ames, IA 50011, USA

**Keywords:** amino acid, beef cattle, lactation, late gestation

## Abstract

The objective was to evaluate the effects of peripartum supplementation of a methionine hydroxy analogue (**MHA**) to primiparous, spring-calving beef females on dam and progeny performance. Angus heifers (*n* = 60) were blocked by expected parturition date, stratified by body weight (**BW**) and body condition score (**BCS**), and randomized to 1 of 15 pens. Pens were randomly assigned to 1 of 3 dietary treatments: a basal diet supplemented with 0 (**M0**), 15 (**M15**), or 30 (**M30**) g/animal/d of MHA (provided as MFP feed supplement, Novus International Inc., St. Charles, MO). Diets were fed from 45 ± 13 (SD) d pre-calving through 81 ± 13 d postpartum (**DPP**), after which all cow–calf pairs were managed as a single group on pasture until weaning (199 ± 13 DPP). Dam BW, BCS, and blood samples were taken at 6 predetermined timepoints. Progeny data were collected at birth, 2 intermediate timepoints, and at weaning. Milk samples were collected for composition analysis at 7 ± 2 DPP and at 55 ± 5 DPP. Serial progesterone samples were analyzed to establish resumption of cyclicity, and ultrasonography was performed at 55 ± 5 DPP to evaluate ovarian function. Cows were bred via artificial insemination at 82 ± 13 DPP and subsequently exposed to bulls for a 55-d breeding season. Pen was the experimental unit, and preplanned orthogonal contrasts were tested (linear effect and M0 vs. M15 + M30). Dam BW and BCS were not affected by treatment (*P* ≥ 0.29) throughout the study. Week 1 milk fat concentration increased linearly (*P* = 0.05) and total solids tended to increase linearly (*P* = 0.07) as MHA increased; however, no other milk components were affected (*P* ≥ 0.16). Treatment did not affect (*P* ≥ 0.16) dam reproductive parameters or progeny growth from birth until weaning. Post-calving, circulating methionine equivalents tended to linearly increase (*P *= 0.10) with increasing MHA supplementation. At breeding, plasma urea N linearly decreased (*P *= 0.03) with increased supplementation of MHA, and plasma non-esterified fatty acids were less (*P *= 0.04) in MHA-supplemented dams compared with dams receiving no MHA. Maternal circulating glucose, glutathione peroxidase, and thiobarbituric acid-reactive substances were not affected (*P* ≥ 0.15) by treatment at any point. These data indicate that peripartum supplementation of MHA may increase milk fat composition shortly after calving, but MHA supplementation did not improve progeny growth or dam reproductive performance in the current study.

## INTRODUCTION

Proper peripartum nutrition is necessary for maximizing beef cattle performance, not only for fetal development, milk production, and progeny growth but also for repairing the reproductive tract and preparing to establish the subsequent pregnancy. Nutrient requirements increase exponentially during late gestation concomitant with rapid fetal growth and then remain high or even increase during early lactation. Primiparous females face the greatest challenge during this time, not only needing to meet their requirements for maintenance, pregnancy, and lactation but also their own growth requirements. Methionine is often considered the first limiting or co-limiting amino acid in cattle for growth (Richardson and Hatfield, 1978), pregnancy (Waterman et al., 2007), and lactation (Schwab et al., 1976; Rulquin and Delaby, 1997). Therefore, providing an increased postruminal supply of methionine may be advantageous during times of high nutrient requirements and potential nutritional stress, such as the case in the peripartum primiparous female.

Providing free amino acid sources becomes wasteful due to a substantial proportion being degraded in the rumen and not reaching the small intestine intact. Ruminally protected forms, such as amino acids encapsulated with lipids or pH-sensitive materials and synthesized amino acid analogues, have been devised for this reason (NRC, 2001). The most widely studied methionine analogue is 2-hydroxy-4-(methylthio)butanoic acid or methionine hydroxy analogue (**MHA**). In a meta-analysis of lactational performance in dairy cows, Zanton et al. (2014) illustrated that MHA promoted increases in milk fat yield as well as numerical increases in both milk yield and milk protein yield. Additionally, Hersom et al. (2009) reported that feeding 15 g/d of MHA to growing beef heifers improved average daily gain (**ADG**) and reproductive tract scores.

The objectives of this study were to evaluate dam and progeny performance of primiparous females fed to meet their nutrient requirements and supplemented with MHA during the last month of gestation and continuing until subsequent breeding. These measures included maternal performance, milk composition, ovarian function, reproductive success, metabolic status, and oxidative stress in the dam and size at birth, pre-weaning growth, and metabolic status in the progeny. The hypothesis was that maternal supplementation of MHA would result in improved calf growth through increased nutrient concentrations in milk without hindering reproductive success.

## MATERIALS AND METHODS

### Animals and Diets

All research was conducted in accordance with procedures approved by the Iowa State University Institutional Animal Care and Use Committee (protocol 11-16-8392-B) at the McNay Memorial Research and Demonstration Farm in Chariton, Iowa from February to October 2017. Angus heifers of similar genetic background (*n* = 60; body weight [**BW**] = 452 ± 28 [SD] kg; body condition score [**BCS**] = 6.0 ± 0.5) and managed as a single group from weaning until treatment initiation were used in a randomized complete block design to elucidate the effects of MHA during late gestation and early lactation. Heifers were blocked by expected parturition date using ultrasonographic fetal age estimates (artificial insemination [**AI**]-bred [average calving date: 3/13/2017] or bull-bred [average calving date: 4/5/2017]), stratified by BW and BCS, and randomized to 1 of 15 pens (4 females per pen). Pens were randomly assigned to 1 of 3 dietary treatments (5 pens per treatment) consisting of a basal diet supplemented with 0 (**M0**), 15 (**M15**), or 30 (**M30**) g/animal/d of MHA (provided as MFP feed supplement, Novus International Inc.). The initiation of treatments began 45 ± 13 d prior to the average parturition date and continued until breeding at 81 ± 13 d postpartum. The basal diet (Table 1) was a limit-fed total mixed ration designed to meet or exceed nutrient requirements for late gestation and early lactation (NASEM, 2016) targeting a prepartum maternal ADG of 0.45 kg and the maintenance of postpartum BCS until the end of supplementation. Diet formulation was done using individual ingredient nutrient composition obtained from near-infrared spectroscopy analysis (Dairyland Laboratories Inc., Arcadia, WI). By the beginning of the sixth week of the study, all 15 pens had become fully accustomed to the basal diet and were consuming all the feed offered. The targeted nutrient requirements for the postpartum period remained the same across the 3 periods; however, the diet was changed due to ingredient availability and inclusion of mineral with elevated magnesium concentration prior to cows being placed on pasture.

**Table 1.** Ingredients and nutrient composition of diets fed to dams from 45 ± 13 d pre-calving through 81 ± 13 d postpartum

	Stage of production^1^			
Item	Prepartum	Postpartum 1	Postpartum 2	Postpartum 3
Ingredient, % of diet DM				
Corn silage	36.7	44.4	48.5	54.3
Ground grass hay	54.7	41.0	21.5	17.1
Modified wet distillers grains with solubles	3.6	9.4	–	–
Dried distillers grains with solubles	–	–	24.5	22.2
Ground corn^2^	3.9	4.1	4.3	4.3
Co-product mineral^3^	1.1	1.2	1.2	–
Hi-mag mineral^4^	–	–	–	1.2
Limestone	–	–	–	1.0
Estimated DM intake^5^				
kg DM/animal/d	9.77	9.66	9.36	9.88
Diet nutrient composition^6^				
CP, % DM	9.0	10.1	11.4	10.7
NDF, % DM	50.8	47.2	39.8	38.8
ADF, % DM	31.9	29.3	23.9	23.5
Lignin, % DM	4.6	4.3	4.3	4.1
Starch, % DM	17.6	20.2	22.2	23.9
Fat, % DM	2.8	3.4	4.1	4.0
Ash, % DM	7.3	6.9	6.1	6.9
TDN, % DM	62.0	65.8	73.4	73.4
Ca, % DM	0.58	0.54	0.48	0.58
P, % DM	0.27	0.29	0.34	0.35
Dry matter, %	60.7	55.6	56.8	54.5

^1^Prepartum diet fed from 45 ± 13 d pre-calving to 4 ± 13 d pre-calving, postpartum 1 diet fed from 3 ± 13 d pre-calving to 30 ± 13 d postpartum, postpartum 2 diet fed from 31 ± 13 d postpartum to 52 ± 13 d postpartum, postpartum 3 diet fed from 53 ± 13 d postpartum to 81 ± 13 d postpartum.

^2^Ground corn served as the carrier blended with 5.5% soy oil as a binder to deliver methionine hydroxy analog (MHA; provided as MFP feed supplement, Novus International Inc.) at 0 (M0), 15 (M15), or 30 (M30) g/animal/d.

^3^Co-product mineral supplement contained: 13.5% Salt, 22.7% Ca, 0.05% P, 1.0% Mg, 0.15% K, 1450 mg/kg Cu, 4950 mg/kg Mn, 26.4 mg/kg Se, 4750 mg/kg Zn, 837800 IU/kg Vitamin A, 220500 IU/kg Vitamin D3, and 827 IU/kg Vitamin E.

^4^Hi-mag mineral supplement contained: 13.5% Salt, 7.2% Ca, 3.75% P, 12.0% Mg, 0.1% K, 1450 mg/kg Cu, 4950 mg/kg Mn, 26.4 mg/kg Se, 4750 mg/kg Zn, 523600 IU/kg Vitamin A, 41900 IU/kg Vitamin D3, and 419 IU/kg Vitamin E.

^5^Calculated as DM delivered to each pen (subtracted feed refusal DM prepartum) divided by the number of head days.

^6^Ingredient composition was based on near-infrared spectroscopy analysis values of corn silage, ground hay, modified distillers, and dried distillers and NASEM (2016) or label values of other ingredients.

The basal diet was delivered in concrete fence-line bunks once daily at approximately 0800 hours. The corn carrier containing MHA was top dressed onto the basal diet by hand immediately behind the feed wagon at a rate of 0.45 kg/animal/d so that dams received 0, 15, or 30 g daily of MHA, based on treatment. During the treatment period, cattle were housed in a gable style barn with adjoining concrete outdoor lots with > 91 cm of bunk space and > 24 m^2^ of space per cow–calf pair.

At the termination of treatments, all cow–calf pairs were commingled and managed as a singular group on tall fescue-based pasture until weaning (199 ± 13 d postpartum). During this grazing period, no supplementation was offered other than free choice mineral and vitamin supplementation.

### Dam Performance

Initial BW was determined as the average preprandial BW measured on the day prior to and the day of treatment initiation (46 and 45 ± 13 d pre-calving), with identical methodology at the initiation of synchronization of ovulation (73 and 74 ± 13 d postpartum) to determine overall treatment effects without differences in gut fill associated with estrus. A single pre-calving BW was taken at 18 ± 13 d prior to average parturition date, and a single post-calving BW collection occurred at 7 ± 2 d postpartum. A BCS assessment was conducted according to Wagner et al. (1988; 1 = emaciated, 9 = obese) by the same trained investigator at each of the 6 timepoints. For data analysis, non-gravid prepartum dam BW was calculated by subtracting the gravid uterine weight using the following formula (NASEM, 2016) for pregnant heifers:


$$\begin{array}{l} CW\;=\;CBW\;\times \;0.01828\;\times e^{(0.02\;\times \;DP\;-\;0.0000143\;\times \;DP^{2})}, \end{array}$$

where CW = conceptus weight, kg; CBW = calf body weight, kg; and DP = days pregnant. For females bred by natural service, days pregnant were estimated based on ultrasonographic fetal age estimates.

While on pasture, dam BW and BCS were collected again at the first pregnancy detection (117 ± 13 d postpartum) and at weaning (199 ± 13 d postpartum).

Ten females were removed due to lack of complete study data (because of late term abortions, stillbirths, neonatal calf death, dam temperament, and udder issues); however, there was no evidence to suggest those problems resulted from treatment (M0 = 3 removals, M15 = 4 removals, and M30 = 3 removals). When cattle were removed from their pen, the feed and supplement delivery to that pen were adjusted accordingly.

### Calf Management and Data Collection

Calf sex and BW were recorded within 24 h of birth. At 7 ± 2 d of age, progeny body measurements were taken using a tape measure, which included biparietal width (distance from the distal corner of 1 eye to the distal corner of the other eye), front cannon length (length of the metacarpus from the proximal to distal epiphysis), and body length (length from the dorsal edge of the scapula down the spine to the posterior point of the ischium). Calf BW was measured again at 55 ± 5 d of age (week 8 milk sampling), 117 ± 12 d of age (first pregnancy detection), and 199 ± 12 d of age (weaning).

As progeny became older, they were observed consuming the dams’ ration because creep feed was not available. As a result, from 61 ± 13 d of age until 81 ± 13 d of age, 0.91 kg of additional dry matter (**DM**) per calf was added to the daily feed delivery of each pen, in identical proportions to the cow diet, to account for the feed consumption of calves and to prevent hindrance of targeted dam performance.

### Milk Composition

Two separate milk samples were collected from all cows at 7 ± 2 d postpartum (week 1) and 55 ± 5 d postpartum (week 8). Milk samples were taken preprandial, with the week 1 sample collected on the Thursday falling between 3 and 9 d postpartum to ensure sufficient time for all colostrum to be removed from the mammary gland. The week 8 sample was taken on the Thursday that was closest to 60 d postpartum. The rear right quarter of each cow’s udder was cleaned and then milked completely by hand. If there was not enough sample volume obtained, the same procedure was administered to the rear left quarter, and the collections were mixed. Milk samples were transferred to plastic tubes (provided by Dairy Lab Services, Dubuque, IA) containing potassium dichromate preservative and immediately frozen. Frozen milk samples were transported to Dairy Lab Services and milk composition was analyzed via infrared instrumentation. The 2 samples collected from each cow were analyzed separately.

### Reproductive Analyses

At the weekly data collection timepoint closest to 60 d postpartum (55 ± 5 d postpartum), cows were subjected to transrectal ultrasonography (Ibex Portable Ultrasound, variable MHz linear array transducer, E.I. Medical Imaging, Loveland, CO) for determination of antral follicle count (**AFC**), ovarian length, and ovarian height. Ultrasound examinations were performed chute-side by the same trained investigator. All follicles visible to the naked eye (>3 mm) were counted on each ovary in real time. The AFC of each ovary was recorded separately. Additionally, a clear freeze frame of the whole ovary was used along with the caliper function of the ultrasound machine to determine the ovarian height and length. Both ovaries were measured separately; however, statistical analysis was performed only on the average height and length of the 2 ovaries combined. Cows were not synchronized prior to ovarian ultrasonography, resulting in cows on random and unquantified days of their estrous cycle.

At 74 ± 13 d postpartum, all cows were synchronized for ovulation using the 5-d CO-Synch + controlled internal drug release (**CIDR**) protocol. At CIDR removal, an Estrotect heat detection aid (Rockway Inc., Spring Valley, WI) was placed on the tailhead of each cow. Seventy-two hours after CIDR removal, all cows were bred via fixed-time AI (**FTAI**; 82 ± 13 d postpartum). At FTAI, heat detection patches were assessed as an indicator of riding incidences associated with expression of estrus before FTAI (1 = fully activated patch; 2 = >50% activated patch; 3 = <50% activated patch; 4 = patch lost before FTAI). Seven days after FTAI, cows were exposed to a group of 3 Angus bulls for a 55-d breeding season. Conception to FTAI and overall pregnancy rates were determined at 117 ± 13 and 199 ± 13 d postpartum, respectively. Cows that remained in the final analysis of the current study and calved the following year were included in calculating the calving interval between the 2017 and 2018 calving season.

### Plasma and Serum Analyses

Blood samples were collected from all dams at the same 6 major timepoints that dam BW and BCS were collected. On all sampling days, data collection began at approximately 0600 hours, and thus blood samples were taken preprandial during dietary treatments. Blood samples for plasma analysis were collected via jugular venipuncture into two 6-mL tubes containing K2 EDTA (BD Vacutainer, BD, Franklin Lakes, NJ) and immediately placed on ice. Blood samples for serum analysis were collected via jugular venipuncture into 10-mL serum blood collection tubes (BD Vacutainer), and blood was allowed to clot at room temperature prior to being placed on ice for transportation. All blood samples were centrifuged at 2,500 × *g* for 25 min at 4 °C. Plasma and serum were recovered and aliquoted into 5-mL polystyrene tubes and frozen at −20 °C until metabolite analyses were conducted. Progeny blood samples were collected at 7 ± 2 d of age, 55 ± 5 d of age, and 199 ± 12 d of age for determination of plasma urea N and glucose concentrations. Blood samples were collected using an identical protocol for collection and centrifugation as dam blood collection for plasma analysis.

Concentrations of 2-hydroxy-4-(methylthio)butanoic acid, or MHA, and methionine in serum were obtained using a liquid chromatography mass spectrometry method developed internally by Novus International Inc. The MHA calibration was conducted in MHA-free plasma spiked with MHA over a range of 25 to 2000 µg/kg. The relative standard deviation was 1.2% and the percent recovery of the internal standard averaged 104.3%. Methionine calibration was conducted in diluent over a range of 500 to 5000 µg/kg. The relative standard deviation was 1.3% and the percent recovery of the internal standard averaged 107.0%. Data are reported as concentrations of methionine equivalents in serum which represents the sum of MHA and methionine.

For all plasma analyses, samples were read in duplicate in 96-well plates on a microplate reader. Plasma samples were analyzed for urea N with a commercially available kit (Urea Nitrogen Procedure No. 0580, Stanbio Laboratory, Boerne, TX). For dam plasma urea N, the average intraassay coefficient of variation (**CV**) was 3.8% and the interassay CV was 9.0%. For progeny plasma urea N, the average intraassay CV was 4.1% and the interassay CV was 5.2%. Plasma non-esterified fatty acid (**NEFA**) concentration was determined using a commercially available kit (HR Series NEFA-HR (2), Wako Pure Chemical Industries, Ltd., Mountain View, CA). The average intraassay CV was 2.5% and the interassay CV was 1.5%. Plasma samples were analyzed for glucose with a commercially available kit (Autokit Glucose, Wako Pure Chemical Industries, Ltd.). The average intraassay CV was 3.7% and the interassay CV was 3.6%

To determine the effects of MHA supplementation on maternal oxidative stress status, serum samples were evaluated for glutathione peroxidase (**GPX**) and thiobarbituric acid-reactive substance (**TBARS**) concentrations by Novus International Inc. Serum GPX and TBARS were only quantified at initiation of the study, at first pregnancy detection, and at weaning. The concentration of GPX was determined using a bovine glutathione peroxidase ELISA kit (#MBS738198, MyBioSource, San Diego, CA) with an intraassay CV of 7.5%. The concentration of TBARS was determined using a commercially available kit (#100009055, Cayman Chemical, Ann Arbor, MI) with an intraassay CV of 5.0%.

Starting at 7 ± 2 d postpartum, dam blood samples were collected weekly until initiation of synchronization of ovulation (74 ± 13 d postpartum) to determine the duration of the anestrous period. Blood samples were collected using the methodology described above for plasma recovery. Plasma samples were evaluated for progesterone concentration via radioimmunoassay using methodology previously described by Engel et al. (2008). The average intraassay CV was 5.4% and the interassay CV was 3.8%. The average sensitivity across assays was 0.4 ng/mL (95% confidence). Resumption of cyclicity was defined as either 7 d before the date of collection of the first blood sample that contained > 2 ng/mL or 7 d before the first of 2 consecutive blood samples with > 1 ng/mL of plasma progesterone (Gasser et al., 2006). To be considered as having resumed cyclicity, cows must have maintained regular cyclicity through the start of the synchronization of ovulation protocol.

### Statistical Analysis

Effect of treatment for binomial data (calf sex, resumption of cyclicity, post-CIDR estrus, FTAI conception rate, final pregnancy rate) was analyzed using the GLIMMIX procedure of SAS 9.4 (SAS Institute Inc., Cary, NC). The remaining reproductive parameters, milk composition, maternal performance, and progeny performance were analyzed using the MIXED procedure of SAS. For all dependent variables of interest, pen served as the experimental unit, the main fixed effect was treatment, and the random effect was pen. For all maternal variables, days postpartum was initially run as a covariate; however, it was removed from the model when *P* > 0.10 for a specific variable. For all progeny-related variables, the statistical model also included the fixed effect of sex. For progeny BW, day of age was always included as a covariate.

Maternal and progeny metabolite concentrations were analyzed with the MIXED procedure of SAS as repeated measures using the compound symmetry covariance structure (based on having the lowest Bayesian Information Criterion for most variables). The model included the fixed effects of treatment and day, as well as the treatment × day interaction.

Two preplanned orthogonal contrasts were decided a priori: 1) testing a linear effect, and 2) testing the difference between the control treatment (M0) and the 2 treatments containing MHA (M15 and M30). Significance was declared when *P* ≤ 0.05 and tendencies were declared when 0.05 < *P* ≤ 0.10.

## RESULTS

### Dam and Progeny Performance

As designed, dam BW and BCS were not different (*P* ≥ 0.53; Table 2) at the time of treatment initiation. Dam BW and BCS were not affected (*P* ≥ 0.29) by MHA supplementation at any timepoint during either the dietary treatments or until weaning.

**Table 2.** Effect of methionine hydroxy analog (MHA) supplementation fed to primiparous beef females from 45 ± 13 d pre-calving through 81 ± 13 d postpartum on maternal performance

	Treatment^1^					Contrasts	
Item	M0	M15	M30	SEM^2^	*P*-value^3^	Linear	0 vs. MHA^4^
BW, kg^5^							
Initial^6^	458	449	456	7	0.64	0.84	0.53
Pre-calving^6^	516	508	513	9	0.83	0.85	0.65
Post-calving	483	466	479	9	0.38	0.73	0.33
Breeding	481	473	476	10	0.83	0.71	0.58
First pregnancy detection	453	443	442	9	0.63	0.39	0.35
Weaning	441	431	431	7	0.56	0.35	0.29
BCS^5^^,^^7^							
Initial	6.0	6.0	6.1	0.1	0.87	0.76	0.95
Pre-calving	6.0	5.8	6.1	0.1	0.31	0.68	0.68
Post-calving	6.1	6.0	6.0	0.1	0.77	0.60	0.49
Breeding	5.3	4.9	5.4	0.2	0.32	0.84	0.55
First pregnancy detection	4.8	4.4	4.4	0.3	0.55	0.39	0.29
Weaning	4.1	3.8	4.1	0.2	0.53	0.96	0.60

^1^Treatment included a basal diet fed to meet or exceed nutrient requirements and supplemented with MHA (provided as MFP feed supplement, Novus International Inc.) at 0 (M0), 15 (M15), or 30 (M30) g/animal/d.

^2^ *n* = 5 pens.

^3^Probability value for overall treatment *F*-test.

^4^Effect of 0 g/animal/d of MHA vs. all diets containing MHA.

^5^BW = body weight. Sampling timepoints are: Initial (45 ± 13 d pre-calving), pre-calving (18 ± 13 d pre-calving), post-calving (7 ± 2 d postpartum), breeding (74 ± 13 d postpartum), first pregnancy detection (117 ± 13 d postpartum), and weaning (199 ± 13 d postpartum).

^6^Non-gravid pre-calving body weights were determined by subtracting the gravid uterine weight (NASEM, 2016).

^7^BCS = body condition score on scale of 1 to 9 (1 = emaciated, 9 = obese; Wagner et al., 1988).

Progeny birth weight was not affected (*P* ≥ 0.80; Table 3) by treatment. There tended to be a linear decrease (*P *= 0.10) in the biparietal width of offspring at birth with increasing MHA supplementation to dams. Conversely, there was a tendency for increased (*P *= 0.06) front cannon length in progeny born to MHA-supplemented dams compared with those not fed MHA. Offspring body length was not affected (*P* ≥ 0.39) by treatment. Progeny BW was not affected (*P* ≥ 0.31) by maternal MHA supplementation at any of the interim measurements or at weaning. Progeny ADG (calculated from birth to weaning) was not affected (*P* ≥ 0.32) by treatment.

**Table 3.** Effect of methionine hydroxy analog (MHA) supplementation fed to primiparous beef females from 45 ± 13 d pre-calving through 81 ± 13 d postpartum on progeny performance

	Treatment^1^					Contrasts	
Item	M0	M15	M30	SEM^2^	*P*-value^3^	Linear	0 vs. MHA^4^
Sex of calves, % heifers	36.7	31.3	52.9	–	0.43	0.32	0.67
Neonate measurements, cm^5^							
Biparietal width	12.8	12.8	12.3	0.2	0.19	0.10	0.27
Front cannon length	14.5	15.3	15.2	0.3	0.16	0.13	0.06
Body length	57.4	58.0	56.5	0.8	0.38	0.39	0.86
Progeny BW, kg^6^							
Birth	32.2	31.7	31.9	1.3	0.96	0.88	0.80
55 ± 5 d of age	82.7	85.6	81.7	3.6	0.71	0.84	0.82
117 ± 12 d of age	125	128	119	4	0.30	0.33	0.82
Weaning^7^	186	190	179	5	0.32	0.31	0.75
Pre-weaning ADG, kg	0.78	0.79	0.74	0.03	0.36	0.32	0.72

^1^Treatment included a basal diet fed to meet or exceed nutrient requirements and supplemented with MHA (provided as MFP feed supplement, Novus International Inc.) at 0 (M0), 15 (M15), or 30 (M30) g/animal/d.

^2^ *n* = 5 pens.

^3^Probability value for overall treatment *F*-test.

^4^Effect of 0 g/animal/d of MHA vs. all diets containing MHA.

^5^Measured at 7 ± 2 d of age.

^6^Day of age used as covariate in the statistical model.

^7^Calves were weaned at 199 ± 12 d of age.

### Milk Composition

At the week 1 (7 ± 2 d postpartum) milk sampling, milk fat linearly increased (*P *= 0.05; Table 4) with increasing MHA supplementation and milk fat tended to be greater (*P* = 0.08) in cows supplemented with MHA compared with dams receiving no MHA. Additionally, there was a tendency for a linear increase (*P* = 0.07) in percent milk total solids as dams received greater concentration of MHA. Milk protein, lactose, other solids, and urea N were not affected (*P* ≥ 0.25) due to dietary treatment at the week 1 measurement. At the week 8 (55 ± 5 d postpartum) milk collection, milk composition was not affected (*P* ≥ 0.16) by treatment.

**Table 4.** Effect of methionine hydroxy analog (MHA) supplementation fed to primiparous beef females from 45 ± 13 d pre-calving through 81 ± 13 d postpartum on milk composition

	Treatment^1^					Contrasts	
Item	M0	M15	M30	SEM^2^	*P*-value^3^	Linear	0 vs. MHA^4^
Week 1^5^							
Milk fat, %	2.7	3.5	4.3	0.5	0.12	0.05	0.08
Milk protein, %	3.6	3.7	4.5	0.6	0.52	0.32	0.54
Milk lactose, %	4.4	4.4	4.2	0.1	0.53	0.41	0.74
Milk other solids, %	5.3	5.4	5.2	0.1	0.58	0.48	0.81
Milk total solids, %	11.7	12.7	13.9	0.8	0.18	0.07	0.13
Milk urea N, mg/dL	8.0	8.5	9.2	0.7	0.50	0.25	0.33
Week 8^6^							
Milk fat, %	3.1	3.2	4.1	0.5	0.34	0.19	0.38
Milk protein, %	2.9	2.8	2.9	0.1	0.35	0.94	0.43
Milk lactose, %	5.0	4.9	4.9	0.1	0.77	0.52	0.48
Milk other solids, %	5.9	5.8	5.8	0.1	0.75	0.53	0.46
Milk total solids, %	11.8	11.8	12.8	0.4	0.24	0.16	0.42
Milk urea N, mg/dL	10.3	9.3	10.3	0.6	0.42	0.99	0.50

^1^Treatment included a basal diet fed to meet or exceed nutrient requirements and supplemented with MHA (provided as MFP feed supplement, Novus International Inc.) at 0 (M0), 15 (M15), or 30 (M30) g/animal/d.

^2^ *n* = 5 pens.

^3^Probability value for overall treatment *F*-test.

^4^Effect of 0 g/animal/d of MHA vs. all diets containing MHA.

^5^Samples collected at 7 ± 2 d in milk.

^6^Samples collected at 55 ± 5 d in milk.

### Reproductive Analyses

The percentage of primiparous females that had resumed cyclicity by the beginning of the synchronization of ovulation protocol was not affected (*P* ≥ 0.25; Table 5) by treatment. Additionally, for those cows that had resumed cyclicity, the anestrous period (measured as the number of days between calving and first estrus) was not affected (*P* ≥ 0.16) by treatment.

**Table 5.** Effect of methionine hydroxy analog (MHA) supplementation fed to primiparous beef females from 45 ± 13 d pre-calving through 81 ± 13 d postpartum on ovarian function and reproductive performance

	Treatment^1^					Contrasts	
Item	M0	M15	M30	SEM^2^	*P*-value^3^	Linear	0 vs. MHA^4^
Ovarian ultrasonography^5^							
Left antral follicles	8.5	8.9	7.2	0.8	0.26	0.24	0.66
Right antral follicles	9.3	10.6	8.5	1.0	0.32	0.55	0.83
Total antral follicles	17.8	19.6	15.6	1.4	0.18	0.29	0.93
Ovarian length, mm	25.8	26.9	25.3	1.1	0.60	0.79	0.80
Ovarian height, mm	14.7	14.9	14.1	0.6	0.60	0.47	0.78
Anestrous period, d	53.7	47.2	41.7	6.2	0.34	0.16	0.17
Cyclic, %^6^	56.7	61.7	41.7	–	0.35	0.25	0.57
Patch score at FTAI^7^	1.34	1.93	1.80	0.32	0.42	0.32	0.21
Patch score 1 or 2, %	86.7	58.3	63.3	–	0.72	0.60	0.46
FTAI conception rate, %^8^	66.7	41.7	53.3	–	0.35	0.51	0.23
Final pregnancy rate, %^9^	95.0	81.7	81.7	–	0.64	0.41	0.36
Calving interval, d	368	371	376	6	0.60	0.33	0.43

^1^Treatment included a basal diet fed to meet or exceed nutrient requirements and supplemented with MHA (provided as MFP feed supplement, Novus International Inc.) at 0 (M0), 15 (M15), or 30 (M30) g/animal/d.

^2^ *n* = 5 pens.

^3^Probability value for overall treatment *F*-test.

^4^Effect of 0 g/animal/d of MHA vs. all diets containing MHA.

^5^Ovarian ultrasonography performed at 55 ± 5 d postpartum.

^6^Cyclic denotes percentage of females exhibiting regular estrous cycles before initiation of the synchronization of ovulation protocol.

^7^FTAI = fixed-time artificial insemination. Patch scores were recorded at FTAI, 1 = fully activated patch; 2 = >50% activated patch; 3 = inactivated patch; 4 = patch lost before FTAI.

^8^Number of confirmed pregnant cows at 35 d post-AI / number of cows inseminated.

^9^Number of confirmed pregnant cows at 56 d post-bull removal / number of cows exposed.

When ovarian ultrasonography was performed at 55 ± 5 d postpartum, AFC for each ovary and total AFC were not affected (*P* ≥ 0.24; Table 5) due to MHA supplementation. Additionally, ovarian height and ovarian length were not affected (*P* ≥ 0.47) by treatment.

Patch scores taken at FTAI were not affected (*P* ≥ 0.21; Table 5) by treatment, nor did MHA treatment affect the proportion of patch scores 1 or 2, which signified physical expression of estrus. Conception rate to FTAI and overall breeding season pregnancy rate was not affected (*P *≥ 0.23) by dietary treatment. Additionally, the calving interval was not affected (*P* ≥ 0.33; Table 5) by treatment.

### Dam and Progeny Metabolite Analyses

The effect of day was significant (*P* < 0.001) for all dam metabolite concentrations but will not be discussed for brevity. There tended to be a treatment × day interaction (*P *= 0.06; Table 6) for concentration of serum methionine equivalents. At the post-calving timepoint, serum methionine equivalents tended to linearly increase (*P *= 0.10) with increasing supplementation of MHA. Concentration of serum methionine equivalents was not affected (*P* ≥ 0.28) by MHA supplementation at treatment initiation or after dietary treatments ended. Concentration of plasma urea N was not affected by the treatment × day interaction (*P *= 0.31; Table 6) or by treatment (*P *= 0.25). Plasma urea N linearly decreased (*P *= 0.03) at breeding (the conclusion of dietary treatments) with increased supplementation of MHA, and MHA-supplemented dams tended to have lower (*P* = 0.07) plasma urea N compared with control dams. There was a treatment × day interaction (*P *= 0.03; Table 6) for plasma NEFA concentration. At the conclusion of dietary treatments, plasma NEFA were lower (*P *= 0.04) in cattle supplemented with MHA compared with dams receiving no MHA, and plasma NEFA tended to linearly decrease (*P* = 0.06) with increased MHA supplementation. Plasma NEFA were not affected due to treatment at any other timepoint (*P* ≥ 0.22). Plasma glucose concentration was not affected by the treatment × day interaction (*P *= 0.25; Table 6) or by treatment (*P *= 0.88). Serum glutathione peroxidase activity and TBARS concentrations were not affected by the treatment × day interaction (*P *≥ 0.81; Table 6) or by treatment (*P *≥ 0.64).

**Table 6.** Effect of methionine hydroxy analog (MHA) supplementation fed to primiparous beef females from 45 ± 13 d pre-calving through 81 ± 13 d postpartum on dam blood metabolites

	Treatment^1^					Contrasts	
Item	M0	M15	M30	SEM^2^	*P*-value^3^	Linear	0 vs. MHA^4^
Methionine equivalents, µg/mL^5^^,^^6^					0.06		
Initial	2.71	2.92	2.94	0.16		0.33	0.28
Post-calving	4.26	4.23	4.66	0.16		0.10	0.37
First pregnancy detection	3.53	3.23	3.41	0.18		0.64	0.35
Weaning	2.93	3.27	2.98	0.16		0.81	0.34
Plasma urea N, mg/dL^5^					0.31		
Initial	11.4	11.2	11.4	0.6		0.92	0.84
Pre-calving	5.9	6.5	6.1	0.3		0.58	0.32
Post-calving	6.2	6.7	6.0	0.3		0.72	0.68
Breeding	14.9	14.0	12.6	0.7		0.03	0.07
First pregnancy detection	20.3	21.1	19.5	0.6		0.38	0.96
Weaning	26.4	26.1	25.2	0.8		0.29	0.44
Non-esterified fatty acids, µEq/L^5^					0.03		
Initial	437	433	419	39		0.74	0.82
Pre-calving	288	314	325	28		0.37	0.38
Post-calving	360	484	362	53		0.99	0.34
Breeding	591	458	446	52		0.06	0.04
First pregnancy detection	714	619	623	60		0.29	0.22
Weaning	441	381	484	34		0.38	0.85
Glucose, mg/dL^5^					0.25		
Initial	81.6	82.7	79.9	2.4		0.61	0.91
Pre-calving	74.9	77.2	72.8	2.4		0.55	0.96
Post-calving	78.6	78.0	82.1	2.8		0.39	0.68
Breeding	77.5	80.7	84.0	3.1		0.15	0.21
First pregnancy detection	77.1	76.8	74.8	1.9		0.40	0.58
Weaning	75.7	77.6	76.3	1.6		0.77	0.51
Glutathione peroxidase, ng/mL^5^					0.81		
Initial	123	140	139	18		0.55	0.47
First pregnancy detection	149	184	165	19		0.56	0.29
Weaning	151	170	168	21		0.58	0.51
TBARS, µM^5^^,^^7^					0.86		
Initial	6.8	6.8	6.9	0.2		0.76	0.79
First pregnancy detection	5.6	5.6	5.5	0.2		0.85	0.93
Weaning	5.5	5.9	6.0	0.3		0.25	0.24

^1^Treatment included a basal diet fed to meet or exceed nutrient requirements and supplemented with MHA (provided as MFP feed supplement, Novus International Inc.) at 0 (M0), 15 (M15), or 30 (M30) g/animal/d.

^2^ *n* = 5 pens.

^3^Probability value for overall treatment × day interaction, there was no main effect of treatment (*P* > 0.10) for any variable.

^4^Effect of 0 g/animal/d of MHA vs. all diets containing MHA.

^5^Sampling timepoints are: Initial (45 ± 13 d pre-calving), pre-calving (18 ± 13 d pre-calving), post-calving (7 ± 2 d postpartum), breeding (74 ± 13 d postpartum), first pregnancy detection (117 ± 13 d postpartum), and weaning (199 ± 13 d postpartum).

^6^Sum of MHA and methionine.

^7^TBARS = thiobarbituric acid-reactive substances.

The effect of day was significant (*P* < 0.001) for all progeny metabolite concentrations but will not be discussed for brevity. There was a treatment × day interaction for progeny plasma glucose concentration (*P *= 0.04; Table 7), but the chosen contrasts were not affected (*P* ≥ 0.44) at any timepoint. Progeny plasma urea N concentration was not affected by the treatment × day interaction (*P *= 0.82) or by treatment (*P *= 0.57).

**Table 7.** Effect of methionine hydroxy analog (MHA) supplementation fed to primiparous beef females from 45 ± 13 d pre-calving through 81 ± 13 d postpartum on progeny glucose and blood urea N concentrations

	Treatment^1^					Contrasts	
Item	M0	M15	M30	SEM^2^	*P*-value^3^	Linear	0 vs. MHA^4^
Glucose, mg/dL					0.04		
7 ± 2 d of age	127	132	125	5		0.78	0.84
55 ± 5 d of age	113	106	114	3		0.77	0.39
Weaning^5^	86	88	84	2		0.44	0.96
Plasma urea N, mg/dL					0.82		
7 ± 2 d of age	12.3	10.8	10.6	1.1		0.26	0.23
55 ± 5 d of age	9.0	8.8	8.9	0.6		0.88	0.85
Weaning^5^	20.0	19.4	19.8	0.8		0.86	0.66

^1^Treatment included a basal diet fed to meet or exceed nutrient requirements and supplemented with MHA (provided as MFP feed supplement, Novus International Inc.) at 0 (M0), 15 (M15), or 30 (M30) g/animal/d.

^2^ *n* = 5 pens.

^3^Probability value for overall treatment × day interaction, there was no main effect of treatment (*P* > 0.10) for any variable.

^4^Effect of 0 g/animal/d of MHA vs. all diets containing MHA.

^5^Calves were weaned at 199 ± 12 d of age.

## DISCUSSION

### Dam and Progeny Performance

The objective of this study was to measure various performance parameters in late gestation and early lactation primiparous beef females when a basal diet formulated to meet daily dietary nutrient requirements for the production stage was supplemented with increasing concentration of MHA. Maternal BW and BCS were not affected by MHA supplementation during or after the dietary treatments. The late gestational diet was formulated to allow for a maternal ADG of 0.45 kg so females would reach 85% of their estimated mature BW by calving. When averaged across all treatments, the maternal ADG achieved between the initial and post-calving timepoints was 0.42 kg. The abrupt increase in maternal BW at the pre-calving timepoint compared with the timepoints on either side of it likely was partially explained by differences in rumen fill. During early lactation, the basal diet that was formulated to meet or exceed nutrient requirements (NASEM, 2016) underestimated the nutrient needs of lactating dams as they lost on average 0.79 BCS prior to breeding. When grazing on pasture, after dietary treatments, it was evident that cattle were not consuming enough nutrients to meet their requirements for maintenance, growth, lactation, and subsequent pregnancy. While this is not completely unexpected for first parity females grazing cool-season based pastures in the summer, it was exacerbated by a particularly dry season. From breeding until weaning, dams lost an average of 42 kg BW and 1.18 BCS, mobilizing adipose stores (elevated NEFA) and catabolizing tissue protein (elevated plasma urea N) to meet requirements. Additionally, it appears that when dams were not meeting their nutrient requirements, MHA supplementation did not mitigate the negative effects on dam BW and BCS loss during lactation.

Maternal BW and BCS were not affected in fall-calving beef cows grazing tall fescue-based pastures and supplemented with 10 g/d of MHA from 23 d prepartum through 73 d postpartum (Clements et al., 2017). Methionine hydroxy analog supplementation at 15 g/d for approximately the last 60 d of gestation and first 30 d of lactation did not affect maternal BW or BCS in *B. indicus*-influenced heifers (Moriel et al., 2020) or cows (Palmer et al., 2020) grazing bahiagrass pastures. Studies in crossbred beef cows supplemented with MHA during late gestation (Collins et al., 2019) or during the periconceptional period (Silva et al., 2021) also reported no changes in maternal BW. The consistent lack of effects on maternal performance due to MHA supplementation, even in our M30 treatment that provided at least 50% more MHA than the previous studies, would suggest that a physiologically impactful dose was not reached or that cattle were not methionine deficient in those studies. Previous studies supplementing dairy cattle with rumen-protected methionine (**RPMet**) products likewise observed no effect on BW and BCS due to treatment during the peripartum period (Toledo et al., 2021) or strictly during lactation (Socha et al., 2005).

Methionine hydroxy analog supplementation during late gestation did not affect calf birth weight, which agrees with other studies in beef heifers (Moriel et al., 2020) and cows (Clements et al., 2017; Collins et al., 2019; Palmer et al., 2020). Periconceptional MHA supplementation did not affect calf birth weight (Silva et al., 2021), but RPMet supplementation for 90 d prior to calving (Moisá et al., 2019) tended to increase calf birth weight in primiparous females. The source and amount of methionine supplemented and the duration and timing of supplementation relative to fetal development milestones are likely explanations for the inconsistent results. Like maternal performance, calf birth weight results would suggest methionine requirements for fetal growth were met by the basal diet during late gestation thus additional MHA did not increase calf birth weight. Protein supplementation and feeding protein in excess of late gestational requirements has even had inconsistent effects on calf birth weight (Stalker et al., 2006; Gunn et al., 2014; Hare et al., 2019; Kennedy et al., 2019); thus, it is not surprising that the minimal difference in maternal dietary supply of nutrients did not affect progeny birth weight in the present study.

In agreement with offspring birth weight, neonatal body length was not affected by MHA supplementation. In 1 study that measured shoulder to rump length (similar to the body length measurement in the current study), it had a weak positive relationship to progeny birth weight (Meyer et al., 2016), while in the current study there was a strong relationship between calf birth weight and body length (data not shown). In the current study, neonatal front cannon length tended to increase in progeny of MHA-supplemented dams while biparietal width tended to linearly decrease with increased maternal MHA supplementation. The mechanism behind the contrasting tendencies for fetal size measurements is unknown.

Pre-weaning progeny performance was not affected by MHA supplementation, which concurs with previous results (Clements et al., 2017; Moriel et al., 2020; Palmer et al., 2020). In Moisá et al. (2019), where calves born to dams supplemented with RPMet were larger at birth, there was still no effect on weaning weights. Periconceptional supplementation of MHA also had no effect on adjusted weaning weights (Silva et al., 2021).

### Milk Composition

Milk fat concentration increased with increasing MHA supplementation at the week 1 sampling in the current study and is likely what accounted for the tendency for total milk solids to also increase. Clements et al. (2017) did not see an effect of peripartum MHA supplementation on milk composition or milk yield at 73 d postpartum. Silva et al. (2021) observed greater milk protein concentration but similar milk fat concentration after periconceptional supplementation of MHA in crossbred beef cows. It should be noted that Clements et al. (2017) and Silva et al. (2021) observed lower percent milk fat and elevated milk urea N concentration across treatments compared with the 55 d postpartum timepoint in the current study. Considering that there were minimal effects on milk composition at the week 1 milk collection and no effect on milk composition at week 8 in the current study, it is not surprising that there was no effect on progeny BW or ADG through weaning. In the current study, milk yield was not measured, and the impact it may have had on progeny performance is unknown. In fact, negative correlations between milk yield and nutrient composition have been demonstrated by Jeffery and Berg (1971) and Rahnefeld et al. (1990). When studies considered correlations between progeny growth and milk yield or milk composition, milk yield is consistently the best variable for explaining differences in progeny performance (Gleddie and Berg, 1968; Chenette and Frahm, 1981; Rahnefeld et al., 1990).

In a meta-analysis, Zanton et al. (2014) compiled MHA studies (46 treatment comparisons) conducted between 1970 and 2011 that evaluated lactational responses in dairy cows supplemented with MHA. Their analysis revealed improvements in milk fat concentration, milk fat yield, milk protein yield, and a numerical increase in milk yield, but no effect on milk protein concentration. Production responses to MHA supplementation have varied greatly across individual studies. Multiple studies have indicated no effect on milk production or milk composition (St. Pierre and Sylvester, 2005; Lapierre et al., 2011; Lee et al., 2015), while others observed increased percent milk fat (Lundquist et al., 1985; Hansen et al., 1991; Baldin et al., 2018), or milk yield (Piepenbrink et al., 2004; Wang et al., 2010). The conflicting results across studies can be attributed to a variety of factors including but not limited to the inclusion rate of MHA, stage of lactation, success or failure in truly meeting the nutrient requirements of lactation, and the variation in metabolism and milk production between dairy and beef cows.

Multiple hypotheses exist for the mechanism in which milk fat increases due to RPMet supplementation. These include alleviating the effects of ruminal biohydrogenation-induced milk fat depression (Baldin et al., 2018), methionine’s role as a methyl donor in the synthesis of choline (Sharma and Erdman, 1988), increased triglycerides and lipoproteins in the blood and subsequent uptake by the udder (Huber et al., 1984), or a stimulating effect on de novo synthesis of short- and medium-chain fatty acids in the mammary gland (Pisulewski et al., 1996).

### Reproductive Analyses

There was no effect of treatment on the percent of cows cycling at the time of breeding in the current study, in agreement with the study of Clements et al. (2017). Rumen-protected methionine supplementation did not affect the percent of dairy cows returning to cyclicity by 49 d in milk (Stangaferro et al., 2021), as determined by progesterone concentration, or the percent of cows responding to a synchronization of estrus protocol (Toledo et al., 2017). Luteinizing hormone (**LH**) concentration in the anterior pituitary must increase in the few weeks after parturition, otherwise inadequate LH pulsatility and concentration will result in the failure of dominant follicles to undergo terminal maturation prior to successful ovulation (Yavas and Walton, 2000). In a study conducted by Grimard et al. (1995), LH pulse frequency at 30 d postpartum was inversely correlated to NEFA concentration. In the current study, cattle that were supplemented with MHA had lower NEFA at the breeding timepoint as well as numerically shorter anestrous periods; however, only about half of all females had resumed cyclicity prior to synchronization. LH pulse frequency and amplitude during the anestrous period was not determined.

In a previous study (Geppert et al., 2017), beef cows fed 150% of metabolizable protein requirements had greater AFC compared with cows fed at 125% of requirements. In contrast, there was no effect of dietary treatments on AFC or ovarian dimensions in the present study. To our knowledge, no previous studies providing MHA supplementation have considered the effect on AFC or ovarian size as secondary reproductive measurements. In the current study, cattle were not synchronized prior to ovarian ultrasonography due to limited labor. However, a repeatability of 0.95 was reported for AFC during the ovulatory and nonovulatory waves of an estrous cycle within a single animal (Burns et al., 2005), despite a sevenfold variation in the average number of antral follicles among the group of cows.

No effect of treatment on FTAI conception rate or final pregnancy rate was observed in the current study, which agrees with other MHA studies (Clements et al., 2017; Moriel et al., 2020; Palmer et al., 2020). Furthermore, periconceptional supplementation of MHA to crossbred beef cows did not affect pregnancy rates after being exposed to bulls for a 75-d breeding season (Silva et al., 2021). The current study likely did not have enough statistical power to detect differences in binomial data; however, similar results have been observed in larger dairy cow studies. In dairy cows, pregnancy per AI was not affected by RPMet supplementation during the peripartum period (Stangaferro et al., 2021) or during lactation (Toledo et al., 2017). Despite this, Toledo et al. (2017) reported that RPMet supplementation reduced early embryonic loss in multiparous but not primiparous dams. Elevated plasma urea N concentration due to excess dietary crude protein has often been implicated as having a negative effect on reproductive success. In the current study, although plasma urea N at breeding exhibited a linear decrease with increasing MHA supplementation, it does not appear that it had any impact on conception rate.

### Dam Metabolite Analyses

Preprandial concentration of serum methionine equivalents (sum of MHA and methionine) tended to linearly increase as MHA supplementation increased at the post-calving timepoint; however, methionine equivalents were not affected before or after the dietary treatments in the current study. Clements et al. (2017) observed greater serum MHA concentration but no effect on serum methionine during MHA supplementation, which resulted in a near tendency for greater methionine equivalents. Moriel et al. (2020) took blood samples 4 h after MHA supplementation and observed greater plasma methionine concentration but did not determine MHA. The timing of blood sampling relative to supplement delivery could account for the differences between studies. Initial work in dairy cows fed MHA identified that ruminal and duodenal concentrations reached peak values at 1 and 3 h post-dosing, respectively, and had returned to baseline concentrations 12 h after dosing with rapid uptake of methionine observed (Koenig et al., 1999). A follow-up study fed 0, 25, or 50 g of MHA and observed serum methionine concentration peak between 3 and 6 h post-dosing for both doses of MHA and return to control values by 9 h and 12 to 24 h for the 25 and 50 g dose, respectively (Koenig et al., 2002).

Interestingly, St. Pierre and Sylvester (2005) performed blood collection at 2 h postprandial and detected no effects on serum methionine; however, they were also delivering dietary treatments and MHA supplementation in twice daily feedings. Moreover, methionine concentration was not affected in multiple other studies when MHA was supplemented at varying doses in lactating dairy cows (Rulquin et al., 2006; Lee et al., 2015). Both enzymes required in the conversion of MHA to L-methionine have been identified in the epithelium of the rumen and omasum (Lobley et al., 2006) and MHA escapes via passive diffusion and paracellular transfer across both organs (McCollum et al., 2000). Hepatic removal in both dairy cows (Lapierre et al., 2011) and sheep (Wester et al., 2006) represented only about 30%, leaving an abundance for post-hepatic tissues. If methionine is truly the first limiting amino acid, it is very plausible that tissue absorption of MHA occurs rapidly, followed by conversion to L-methionine and retention for protein synthesis rather than re-entering systemic blood circulation (Clements et al., 2017). Thus, failure to detect an effect on circulating methionine concentration when MHA is supplemented does not necessarily disprove that MHA is an effective source of methionine to ruminants.

It is widely accepted that substantial increases in dietary crude protein, specifically rumen degradable protein, can elevate plasma urea N concentration. The MFP product is formulated to contain 84% MHA, which is estimated to have a 40% rumen bypass rate (Koenig et al., 2002). In the current study, maternal plasma urea N was not sensitive to the slight protein increase caused by MHA supplementation. In fact, it should be noted that the M30 treatment had the lowest numerical plasma urea N at all postpartum timepoints. Supplementation with RPMet resulted in no change in plasma urea N in lactating dairy cows (St-Pierre and Sylvester, 2005; Michelotti et al., 2021), or in growing beef calves until 6 g of sulfur amino acids were provided (Hersom et al., 2009). When Lee et al. (2015) supplemented dairy cows with MHA, plasma urea N quadratically decreased compared with controls. It could be hypothesized that the reduced plasma urea N concentration resulting from supplementation in the current study occurred because methionine was limiting. Bach et al. (2000) postulated that when methionine is supplied to methionine-limited animals, the liver is better able to utilize all amino acids that otherwise would have been deaminated and entered the urea cycle. Across all treatments, dams in the current study had greater concentration of plasma urea N when grazing on pasture than during the dietary treatments; however, cattle were losing BW during this time, and it is likely they were catabolizing tissue protein which would elevate plasma urea N.

In the current study, no effects of treatment were detected in maternal NEFA concentration until breeding (just prior to termination of dietary treatments), when NEFA were lower in MHA-supplemented cattle. Peripartum MHA supplementation in *B. indicus*-influenced beef cows did not alter NEFA (Palmer et al., 2020). Socha et al. (2008) observed a tendency for decreased NEFA during peak lactation and a quadratic decrease over a range of duodenal infusions of methionine during mid lactation; however, there were no effects observed during early lactation or during an earlier study they conducted (Socha et al., 2005). In contrast, early lactational RPMet supplementation in primiparous and multiparous Holstein cows resulted in elevated NEFA (Michelotti et al., 2021). By supplying a more optimal amino acid balance via methionine supplementation, the liver is more effective in exporting NEFA to be used for the synthesis of very low-density lipoproteins (Socha et al., 2008). Cows consuming inadequate nutrient intake have greater NEFA concentration because of the increased dependence on mobilizing body fat to meet energy needs (Socha et al., 2008). This likely explains the rise in NEFA across treatments at the first data collection while on pasture as cattle had become fully accustomed to receiving a nutrient-dense total mixed ration during supplementation and were abruptly placed on summer pasture that was less nutrient dense.

Glucose concentration was not affected by dietary treatment, agreeing with previous MHA supplementation in *B. indicus*-influenced beef heifers (Moriel et al., 2020) and early lactating dairy cows (Michelotti et al., 2021). Collins et al. (2019) observed greater serum glucose during late gestational RPMet supplementation in beef cows; however, Palmer et al. (2020) noted peripartum MHA supplementation resulted in lower glucose in lactation. Socha et al. (2008) reported that glucose was not affected by methionine infusions during peak or mid lactation; however, a quadratic increase was observed during early lactation. It is not surprising that most studies supplying methionine did not see an effect because methionine is not considered a predominant gluconeogenic amino acid. Glucose concentration are much more correlated to dietary energy intake, and in the current study the treatment diets provided were isocaloric.

In the current study, GPX activity was not affected at any point throughout the study. In the high-producing dairy cow, there is a drastic increase in metabolic activity after parturition and as lactation begins, leading to increased production of reactive oxygen species and concurrent depletion of antioxidants (Sordillo and Aitken, 2009). Considering methionine is involved in glutathione synthesis (the major intracellular antioxidant; Finkelstein, 1990), previous work has found merit in the ability of methionine supplementation to alleviate oxidative stress. Osorio et al. (2014) supplemented periparturient dairy cows with RPMet and observed greater oxygen radical absorbance capacity in supplemented cows compared with controls. In agreement, Zhou et al. (2016) reported increased glutathione concentration in supplemented cows; however, reactive oxygen metabolites remained unchanged. Additionally, when dairy cows were supplemented with RPMet during the periparturient period, cows experienced both increased glutathione as well as lower concentration of reactive oxygen metabolites (Batistel et al., 2018).

Analysis of TBARS was conducted to look at lipid peroxidation and to serve as a secondary indicator for oxidative stress status. Thiobarbituric acid-reactive substance concentration was not affected by treatment at initiation of the study or at either the first pregnancy detection or weaning. To our knowledge, no prior work has been completed studying TBARS in periparturient cattle receiving MHA supplementation.

### Calf Metabolite Analyses

No similar data pertaining to progeny pre-weaning plasma urea N concentration after maternal peripartum supplementation with MHA products have been identified. Increased intake of nitrogenous compounds is usually associated with increased blood urea N; however, in the current study as milk urea N concentration numerically increased at 7 ± 2 d postpartum with MHA supplementation, progeny plasma urea N values collected on the same day decreased with increasing maternal supplementation with MHA.

Progeny glucose concentration was not affected by treatment at any point, which agrees with Xu et al. (2018), who delivered RPMet to dairy cows the last 28 d prepartum. Jacometo et al. (2016) fed RPMet to dairy cows for 21 d prior to calving and observed lower progeny glucose prior to colostrum consumption in calves born to RPMet supplemented dams, but no effects from 24 h after colostrum to 50 d of age.

## CONCLUSION

Based on data from the present study, primiparous females fed a basal diet to meet or exceed nutrient requirements and supplemented with MHA at 15 or 30 g daily during late gestation and early lactation may increase milk fat concentration shortly after parturition and alter maternal circulating metabolites around the time of breeding. Plasma methionine equivalents tended to be greater post-calving due to MHA supplementation; however maternal BW, BCS, and reproductive performance were not affected. Additionally, the increase in milk fat did not translate to improved progeny pre-weaning performance. Methionine hydroxy analog supplementation of females already fed to meet peripartum nutrient requirements saw minimal improvement; however, research on MHA supplementation of females fed poor quality forage or experiencing nutrient restriction, especially primiparous dams, is warranted.
